# Hygienic Treatment of Epilepsy

**Published:** 1858-04

**Authors:** 


### THE HYGIENIC TREATMENT OF EPILEPSY.*

We notice this work, the production of one of our most accomplished physicians, with much satisfaction. There is a clearness of style, a readiness in description, and a logical manner of argument throughout, demanding the best recommendations we can possibly offer. The chapter on the hygienic

* On Epilepsy and Epileptiform Seizures. By E. H. SIEVEKING, M.D. London: Churchill. 1858.

treatment of epilepsy concerns us most in this place. Here Dr. Sieveking lays down rules which may be condensed as follows.

Pure air is necessary for the epileptic.

Water as a beverage, and water as a roborant, is necessary to the epileptic.

The food of the epileptic patient, as a general rule, must be nutritious and copious.

Rest of body and of mind exerts a most beneficial influence in epilepsy.

Dr. Sieveking concludes this chapter as follows:

"Frivolous reading books that exclusively excite the emotions, though in themselves of no immoral tendency—but above all books that are calculated to give an improper stimulus to the sexual feelings—from Lemprière, the bane of schoolboys, to *Don Juan* or *Les Mystères de Paris,* the poison of unripe adolescents, should be sedulously avoided. Those abortions of modern civilisation, full-dressed children's parties, which are got up for the sake of pandering to the love of display and vanity of parents much more than for the amusement of the children, must also be mentioned as things specially to be avoided, when the nervous system shows symptoms of unusual sensibility."

We wish that Dr. Sieveking's admirable admonitions were in the hands of every educated man.

